# Does Timing of Antenatal Care Initiation and the Contents of Care Have Effect on Caesarean Delivery in Ethiopia? Findings from Demographic and Health Survey

**DOI:** 10.1155/2021/7756185

**Published:** 2021-08-09

**Authors:** Getnet Gedefaw, Fikadu Waltengus, Asmamaw Demis

**Affiliations:** ^1^School of Midwifery, College of Health Sciences, Woldia University, P.O.Box: 400, Woldia, Ethiopia; ^2^Department of Midwifery, College of Medicine and Health Sciences, School of Health Science, Bahir Dar University, Bahir Dar, Ethiopia; ^3^School of Nursing, College of Health Sciences, Woldia University, P.O.Box: 400, Woldia, Ethiopia

## Abstract

**Background:**

Antenatal care (ANC) is an important preventive set of core healthcare services through pregnancy. Caesarean deliveries are significantly increasing in many low-, middle-, and high-income countries. However, overuse of the caesarean section service interferes with the quality and cost of the procedure. Hence, this study aimed to assess the effect of timing of first antenatal care initiation and the contents of care on caesarean delivery.

**Methods:**

A population level cross-sectional study was conducted with a total of 4757 study participants. The multivariable analysis was computed using the setup of 3 models.

**Results:**

The rate of caesarean section among women who initiated antenatal care in the first trimester was 1.32% (95% CI = 0.91–4.21). Women initiated antenatal care in the first trimester (AOR = 2.74; 95% CI = 1.49–6.2) and received contents of care (AOR = 1.98; 95% CI = 1.24–3.78])were more likely to have caesarean section delivery as compared to their counterparts.

**Conclusion:**

Caesarean section among women who initiated ANC in the first trimester is low. The finding suggests ANC initiated early (within 16 weeks) can have a positive impact on caesarean section delivery. In addition, being urban residents, primipara women, initiating antenatal care before 16 weeks, received contents of care, and having antenatal care visits three and more increase the odds of having caesarean section. As a result, different obstetric, medical, and surgical complications are detected and managed as early as possible.

## 1. Introduction

Caesarean delivery refers to the delivery of the fetus through a surgical incision made through the abdominal wall (laparotomy) and the uterine wall (hysterotomy). According to the World Health Organization (WHO), caesarean deliveries are the most common surgical procedures in the world, nearly 18 million are performed yearly worldwide [1]. Although caesarean delivery has played a significant role in lowering both unfavorable maternal and fetal outcomes during the past decade, however, caesarean delivery has been associated with a variety of short- and long-term maternal and neonatal complications [2]. Although the rate of caesarean section is significantly increasing, however, it is not associated with improved outcomes for mothers and newborns and deliveries may not lead to better health outcomes for women or neonates who do not require the procedure [3–6].

The provision and uptake of quality and timely antenatal care (ANC) are an essential element of efforts to improve health outcomes for women and newborn babies. Antenatal consultations assist in the early identification and treatment of complications during pregnancy. Women not initiating ANC early may lead to late diagnosis of complications which might have the potential to detrimentally affect maternal and fetus health. As a result, it contributes to maternal mortality, premature labor, and preterm babies and intrauterine deaths are significantly increasing. In addition, late initiation of antenatal care might be responsible for complications such as preeclampsia, anemia, and low birth weight among teenage and unmarried women [7].

Studies showed that maternal age, level of education, wealth, parity, and age at marriage were statistically significantly associated with the timing of initiation of antenatal care attendance [8]. Different studies evidenced that women who started ANC attendance early, received components of care, and attended frequently were more likely to be assisted during childbirth by a skilled attendance compared to those who initiated ANC late and attended only a few visits. When the number of receiving contents of antenatal care visits increases, the woman is expected to escalate women's perception about birth preparedness and complication readiness besides other contents of antenatal care [9].

Early initiation of antenatal care helps health workers to provide timely information and services according to the gestational age and the health condition of both the mother and the fetus. But, mothers who attend antenatal care late miss the opportunity to receive health information and interventions such as early detection of HIV, malaria, and anemia prophylaxis and prevention or management of complications [10].

According to the World Health Organization (WHO), every pregnant woman in developing countries should seek ANC within the first trimester of gestation. While preparing for safe childbirth is an essential part of ANC, the timely initiation of the first ANC visit is an important element. Studies have shown that early ANC attendance (during the first trimester of pregnancy) plays a major role in the early detection and treatment of maternal health problems in pregnancy and serves as a good basis for proper management during and after childbirth [11].

Ethiopia has developed different strategies, programs, and policies to improve maternal health services utilization. Caesarean section deliveries, facility delivery cares, family planning services, antenatal care services, and postnatal care services are provided free of charge [12–15].

Early initiation of antenatal care is more expedient in the role of early detection and management of problems related to pregnancy and preventing adverse complications during pregnancy, although women are still starting antenatal care visits in their late pregnancy [16, 17]. Despite antenatal care reduces maternal and perinatal morbidity and mortality with the help of early detection and treatment of pregnancy-related complications, still the contents of care given for women who attended antenatal care are still low. The likelihood of achieving skilled birth attendant, antenatal care, and institutional delivery is less likely to achieve the national target of reproductive health services. Assessing factors and barriers that affect caesarean delivery are crucial to achieving the national target. More studies, with large sample sizes, are needed to identify the factors associated with caesarean delivery in Ethiopia. Therefore, this study tried to examine the effect of timing of antenatal care visits (initiated antenatal care in the first trimester) and the component of antenatal care services on caesarean section delivery.

## 2. Materials and Methods

### 2.1. Study Area and Design

This research analyzed factors reported by the 2016 Ethiopia Demographic and Health Survey (EDHS) from the fourth version of the survey. We used the 2016 EDHS data, the fourth DHS in Ethiopia, which was collected from January 18 to June 27, 2016, for this analysis. It is a community-based, nationally representative, cross-sectional data collected from all 9 regions and two city administrations [18].

### 2.2. Data and Sampling Procedures

Data for this study were retrieved from the 2016 EDHS, which used a weighted multistage, stratified cluster sampling approach. The 2016 EDHS data employed a two-stage stratified cluster sampling procedure for nine regional states and two city administrations. A total of 645 enumeration areas (202 in urban areas and 443 in rural areas) were selected using a probability proportional to the enumeration size. All women aged 15–49 who were either permanent residents of the selected households or visitors who stayed in the household the night before the survey were eligible to be interviewed for interviewer-administered structured questionnaires. A total of 15,683 women aged 15–49 years were interviewed in the 2016 EDHS, of which 7,590 women had at least one live birth in the last 5 years before the survey [18]. Two thousand eight hundred and thirty three women were excluded from the study as 2818 women did not attend antenatal care at all and 15 women did not know to either attend their antenatal care follow up or not. Overall, the included sample size for this study was 4757 women who had at least one antenatal care during the recent pregnancy.

### 2.3. Measurement of Outcomes

#### 2.3.1. Caesarean Delivery

Women who responded as live births delivered by caesarean section coded as yes “1” and no “0.”

### 2.4. Exposure Measurement

#### 2.4.1. Covariates

Socioeconomic and sociodemographic antenatal care service-related factors, fertility, and reproductive health-related characteristics were included as covariates.

  Socioeconomic and sociodemographic characteristics: region, residence, age of the mother, educational status of the mother, educational status of the husband, religion, marital status, working status of the mother, birth order of the recent child, and wealth index.
  Antenatal care service-related factors: timing of first antenatal care, frequency of antenatal care visit, and content of antenatal care.
  Fertility and reproductive health-related factors: parity, the status of pregnancy (whether wanted or not), age at first birth, number of living children, and birth weight of the recent pregnancy.

### 2.5. Data Processing and Analysis

Data were analyzed using SPSS version 20 statistical software. Complex sample survey (stratified/clustered) sampling designs were used to correctly calculate unequal probabilities of selection with weighted data. Rao–Scott chi-square was used to examine the bivariate associations between each covariate and caesarean delivery via adjusting complex survey sampling. Weighting was used to correct for nonresponse and disproportionate sampling. Both bivariate and multivariable logistic regression analyses were performed to identify the impact of timing of antenatal care and content of care on caesarean delivery. Both descriptive and analytical statistics were calculated. Covariates in the bivariate logistic regression with a *p* value of less than 0.25 were a candidate to be fitted into the multivariable logistic regression to control the possible effects of confounders and assess the association between caesarean section and timing of antenatal care initiation and contents of care using the control model. In the multivariable analysis, variables with a *p* value < 0.05 were declared as statistically significant with odds ratios with their corresponding 95% confidence intervals (CI). Multicollinearity was checked using the standard error (SE) with a cut-off point of below 2. As a result, we declared that no multicollinearity was existed in this study [18]. Moreover, the association between the main independent variables (timing of antenatal care and received contents of care) with caesarean section delivery was investigated by using binary logistic regression (model 1).

In model 2, variables that demonstrated a significant association (with a *p* value of ≤0.05) in bivariate analysis excluding timing of antenatal care and received contents of care were exported to a multiple logistic regression model for multivariate analysis. Finally, the timing of antenatal care, parity, number of antenatal care visit, and residence were significantly associated with caesarean section deliveries in the final model after controlling all the possible confounders (model 3).

### 2.6. Operational Definition

  Timely initiation of antenatal care: women who receivied antenatal care from a skilled provider for the most recent birth within 3 months of her pregnancy.
  Caesarean section: a surgical procedure involving incision of the walls of the abdomen and uterus for delivery of offspring.
  Content of antenatal care: according to WHO recommendation, women should receive the following core set maternal services: measuring blood pressure, a urine sample taken, a blood sample taken, told about pregnancy complications, tetanus toxoid vaccination, taken drugs for intestinal parasites, and iron tablets/syrup supplementation.

### 2.7. Ethical Approval and Consent to Participate

All the available datasets were obtained from the DHS website (https://dhsprogram.com/); through registering with the DHS website, no ethical approval was required. Institutional Review Board of Woldia University waived the requirement for informed consent for EDHS data. All the authors have no special access privileges to this dataset. To access and get the data, authors should register and log in. While we request the title of the project, the co-author's name and e-mail address and a brief description of the study should be clearly stated. Then, the researchers continue to select the country, dataset, and year of the survey. Within a few days after requesting, the demographic and health survey team will get permission to download the dataset via e-mail of the corresponding author. After permission is stated, the author can log in and select the specific data with the important format the author wants [18].

## 3. Results

### 3.1. Socioeconomic and Sociodemographic Characteristics

Out of the 4757 study participants, more than two-thirds of the mothers were rural residents. Almost all (93.7%) were married or in union. More than half (69%) were orthodox Christians in terms of religion. Regarding the level of education, most of the mothers (54%) did not attend formal education. Given the wealth index, 22.5% of the mothers were in the richest wealth quintile level (Table 1).

### 3.2. Contents of Antenatal Care, and Its Relation with Caesarean Section Deliveries

In this survey, all women who initiated antenatal care follow-up (in the first trimester) were getting routine components of antenatal care services such as blood pressure measurement, urine analysis, blood group and Rh determination, prophylaxis of iron supplementation, and tetanus toxoid vaccination as compared to women who initiated antenatal care services after 16 weeks of gestation. In this study, 2654, pregnant women, were received antenatal care visits in the first trimester of pregnancy in the health facility. Around twenty-two and twenty-one percent of the population size received tetanus toxoid vaccination and their blood pressure was measured, respectively (Table 2).

### 3.3. Obstetrics, Fertility, and Reproductive Health-Related Characteristics

Out of the overall study participants, nearly half of the 2342 (49.2%) pregnant women had initiating antenatal care visits within 16 weeks of pregnancy (early). More than two-thirds of the pregnancy was wanted and then followed by wanted later (Table 3).

After controlling confounders in each model (1, 2, and 3), the timing of antenatal care, parity, contents of antenatal care, number of antenatal care visits, and residence were the factors significantly associated with caesarean section deliveries in the final model.

Women who initiated their antenatal care visit early (within 16 weeks) of pregnancy (AOR = 2.74; 95% CI = 1.49–6.2) were nearly three times more likely to undergo caesarean section deliveries than the women who initiate their antenatal care follow-up after three months of pregnancy.

The odds of having caesarean deliveries (AOR = 2.13; 95% CI = 1.5–3.72) were two times higher among primipara women than multipara women.

Similarly, the chance of having caesarean section deliveries (AOR = 4.78; 95% CI = 2.8–8.75) were nearly four times among urban residents compared to people living in rural areas.

Women having antenatal care visits more than four times (AOR = 2.85; 95% CI = 1.38–5.73) were more likely to have caesarean section deliveries compared to women who had less than and equal to four antenatal visits.

Women who received any contents of antenatal care were (AOR = 1.98; 95% CI = 1.24–3.78) times more likely to have caesarean section as compared to their counterparts (Table 4).

## 4. Discussion

This study result revealed that among women who attended antenatal care visits in the first trimester, around three in a hundred women had caesarean delivery. The rate of caesarean delivery service use among women who initiated antenatal care visits within 16 weeks of pregnancy was (1.32% (95% CI = 0.91–4.21)) which is low as compared to the WHO caesarean section recommendation rate (5–15%).

The rate of caesarean section is lower than the study conducted in different regions of Ethiopia, Addis Ababa [19], Yirga Alem [20], Hawassa [21], Finote Selam [22], and Chiro [23], and Saudi Arabia [24] and Jordan [25]. The possible justification for lowering of caesarean section is the difference of the study population. i.e., considering study participants who were only women who had attended and received contents of antenatal care in the first trimester of pregnancy might reduce the magnitude of caesarean section since this study focused on the impact of early initiation of antenatal care on caesarean delivery. Furthermore, this study showed evidence of women having early initiation of antenatal care and received contents of standared care with qualified professionals have a impact on mode of delivery.

Early initiation of antenatal care has a significant impact on caesarean section delivery preference. This study finding is supported and showed the overall antenatal care was positively associated with caesarean delivery in Jordan [25] and Addis Ababa [26]. Antenatal care is an indicator for timing, number of antenatal visits, and content of services received during antenatal care visits. Women who received adequate antenatal care early or within three months of pregnancy were more likely to have caesarean section delivery compared to those women who initiated their first antenatal care after 16 weeks of pregnancy.

Keeping in view the significance of antenatal care utilization in determining the mode of delivery, the WHO recommends every pregnant woman to have at least four antenatal care visits during her pregnancy. Mothers who received antenatal care more than four times delivered babies by caesarean section. Although the reason for this is still unclear, the risk-avoiding behavior of gynecologists towards women who reported having pregnancy complications could be one of the reasons for this preference for caesarean delivery. Besides the effect of prenatal education on antenatal clients, early initiation of antenatal care is aimed to prevent health risks, early detection of abnormalities, and institution of corrective measures if possible and preparation of both the woman and fetus and to ensure a good start of life for each newborn child [27, 28].

Pregnant women require knowledge on caesarean delivery. The antenatal visit is the right moment to provide counseling related to this mode of delivery. Inadequate antenatal care in visits could have influenced the caesarean delivery because antenatal care is the opportunity to anticipate macrosomia and induce labor before the expected delivery date and avoid excessive fetal growth [29].

In this study, nearly half of the ANC attended women 49.2% were initiating their antenatal care follow-up within three months of pregnancy. This study finding is higher than the study conducted in Tigray [30] (27.5%) and in the global estimate [31] 24%. This might be because; this study is a national survey population-level analysis which yields a significant representative than a single study. Besides, this inconsistency could be attributed to the scope of the study, the fact that EDHS covered more remote.

Women who resided in urban areas were more than three times likely to have caesarean section deliveries compared to women who resided in rural areas. This factor was also significantly associated with CS delivery in studies conducted in Ethiopia, Mizan Aman [32]. Although women who resided in rural areas may initiate antenatal care, unfortunately, they withdraw their follow-up because of poor counseling on timing, number, and contents of the antenatal care follow-up. As a result, they have no more knowledge on birth preparedness, complication readiness, and mode of delivery as well as where they have to go if they feel labor pain in their home. On the contrary, women who live in urban areas have a chance to access the health facility with good adherence to prenatal counseling on danger signs and they fear labor pain since epidural analgesia during labor is not practiced in our country; therefore, most of the women preferred to have caesarean section deliveries. Even women who are living in urban areas have a high chance of going to private hospitals and caesarean section might be done without being medically indicated with maternal preference.

Caesarean section deliveries are more frequent amongst primipara women. This study finding is supported by the studies conducted in Jordan [25], Vietnam [33], India [34], and Bangladesh [35]. Increasing parity was found to be associated with decreased odds of caesarean section delivery which is consistent with the clinical practice in our country. Nulliparity is associated with a higher rate of induction of labor, cephalopelvic disproportion; as a result, caesarean section delivery is anticipated [36]. Women who received contents of antenatal care were more likely to give birth through caesarean section delivery as compared to their counterparts, since initiating early antenatal care checkups could increase the strength of the relationship between ANC and reductions in neonatal and maternal mortality through received contents of antenatal care [8].

## 5. Conclusion

Caesarean section among women who initiated ANC in the first trimester is low. The finding suggests ANC initiated early (within 16 weeks) can have a positive impact on caesarean section delivery. In addition, being urban residents, primipara women received contents of care, initiating antenatal care before 16 weeks, and having antenatal care visits three and more increase the odds of having caesarean section in Ethiopia. Initiating the antenatal care early can increase the uptake of all contents of antenatal care and enhances the investigation of women further for different obstetric, medical, and surgical complications as early as possible. Avoiding wrong perceptions about the timing of ANC is related to the lack of knowledge about the benefit of early antenatal care and cultural and traditional beliefs related to health care-seeking practices during pregnancy. As a result, more efforts should be taken to increase the knowledge of mothers about the early initiation of antenatal care.

### 5.1. Limitation

The temporal cause and effect relationship might not be possible due to the natural effect of cross-sectional study design.

## Figures and Tables

**Table 1:** Socioeconomic and sociodemographic characteristics (*N* = 4757).

Variable	Frequency	Percent
*Region*		
Tigray	481	10.1
Afar	36	0.7
Amhara	1102	23.2
Oromia	1605	33.8
Somali	117	2.4
Benishangul	56	1.1
SNNPR	1111	23.5
Gambela	15	0.3
Harari	13	0.26
Addis Ababa	192	4.04
Dire Dawa	29	0.6

*Religion*		
Orthodox	2024	42.5
Muslim	1568	33
Others (protestant, Catholic, and traditional)	1165	24.5

*Educational status of the mother*		
No education	2569	54
Primary	1574	33.1
Secondary and above	614	12.9

*Residence*		
Urban	870	18.29
Rural	3887	81.71

*Marital status*		
Married/in union	4470	93.7
Others	287	6.3

*Birth order*		
First	1119	23.5
Second to fourth	2083	43.8
Five or more	1555	32.7

*Maternal age*		
≤19	749	15.8
20–34	3418	71.8
≥35	590	12.4

*Mothers' current working status* ^*∗*^		
Unemployed	1507	31.7
Employed	3250	68.3

*Wealth index*		
Poorest	791	16.6
Poorer	935	19.7
Middle	993	20.8
Richer	966	20.4
Richest	1072	22.5

*Educational level of the husband*		
No education	1810	38
Primary	1813	38.2
Secondary and above	846	17.8

^*∗*^Working currently either in a private, governmental, or nongovernmental organization.

**Table 2:** Percentage of women receiving components/contents of antenatal care (*N* = 4757).

Variable	Frequency	Percent	Odds ratio
*Blood pressure taken*			
Yes	1175	24.7	21.26 (14.09–21.06)
No	3582	75.3	

*Blood sample taken*			
Yes	1303	27.4	20.5 (16.6–24.4)
No	3454	72.6	

*Urine sample taken*			
Yes	3147	66.2	18.68 (15.1–22.27)
No	1610	33.8	

*Told about pregnancy complication*			
Yes	2142	45	17.12 (10.17–15.26)
No	2615	55	

*Iron tablets/syrup supplementation*			
Yes	2855	60	17.58 (14.09–21.06)
No	1889	39.7	
Do not know	13	0.3	

*Tetanus toxoid vaccination*			
Received injection	3466	72.9	22.26 (17.9–26.62)
Not received	1149	24.2	
Do not know	142	2.9	

*Taken drugs for intestinal parasites*			
Yes	364	7.65	6.02 (4.04–8.0)
No	4311	90.6	
Do not know	82	1.75	

**Table 3:** Obstetrics, fertility, and reproductive health-related characteristics (*N* = 4757).

Variable	Frequency	Percent
*Timing of first antenatal care*		
Early (within 16 weeks of pregnancy)	2342	49.2
Late (above 16 weeks of gestation)	2415	50.8

*Wanted status of a child* ^*∗*^		
Wanted then	3612	75.9
Wanted later	821	17.3
Wanted no more	324	0.68

*Parity*		
Multipara^∗∗^	3638	76.5
Primipara	1119	23.5

*Age at first birth*		
≤19	2919	61.4
20–34	1828	38.4
≥35	10	0.2

*Number of children*		
1–2	1960	41.2
3–5	1727	36.3
6 and more	1070	22.5

*Number of antenatal care visits*		
≤4	3536	74.33
>4	1221	25.66

*Birth weight of the recent pregnancy*		
≤2499 gram	259	5.4
2500–3999 gram	797	16.8
≥4000 gram	3701	77.8

^*∗*^Percent distribution of births to women aged 15–49 in the 5 years preceding the survey, including current pregnancies, by planning status of the birth wanted then, wanted later, or not wanted at all. ^∗∗^A woman that has been having had more than one pregnancy resulting in viable offspring.

**Table 4:** Bivariate and multivariable logistic regression

Variable	Caesarean delivery		Model 1	Model 2	Model 3
	No	Yes	COR (95% CI)	AOR (95% CI)	AOR (95% CI)
*Timing of antenatal care*					
Early	2311 (48.6%)	31 (0.7%)	**4.56 (2.5–8.2)**		**2.74 (1.49–6.2)** ^*∗*^
Late	2277 (47.7%)	138 (2.9%)	1		**1**

*Received contents of care*					
Yes	2029	113	**2.54 (1.6–3.97)**		**1.98 (1.24–3.78)** ^*∗*^
No	2559	56	1		**1**

*Birth order*					
One	1048 (22%)	70 (1.5%)		**6.43 (2.8–11.8)**	4.33 (0.72–8.6)
Two to four	2000 (42%)	84 (1.8%)		**3.2 (1.87–7.32)**	2.97 (0.6–5.2)
Five and more	1540 (32.4%)	15 (0.3%)		1	1
*Parity*					
Primipara	1048 (22%)	71 (1.5%)		**2.43 (1.76–3.92)**	**2.13 (1.5–3.72)** ^*∗*^
Multipara	3540 (74.4%)	98 (2.1%)		1	1

*Educational status of the mother*					
No education	2532 (53.2%)	37 (0.8%)		1	1
Primary	1519 (31.9%)	55 (1.2%)		**2.54 (1.34–4.67)**	0.98 (0.67–3.7)
Secondary and above	537 (11.3%)	77 (1.6%)		**9.79 (5.43–17.65)**	1.25 (0.47–2.74)

*Wealth index*					
Poorer	924 (19.4%)	12 (0.2%)		0.88 (0.25–3.16)	0.91 (0.62–3.45)
Poorest	780 (16.4%)	11 (0.2%)		1	1
Middle	977 (20.5%)	16 (0.3%)		1.15 (0.4–3.38)	1.34 (0.67–4.8)
Richer	950 (20%)	15 (0.3%)		1.12 (0.36–3.43)	0.75 (0.12–1.94)
Richest	957 (20.1%)	15 (2.4%)		**8.41 (3.54–19.98)**	1.54 (0.85–3.87)

*Residence*					
Rural	3830 (80.5%)	57 (1.2%)		1	1
Urban	758 (15.9%)	112 (2.3%)		**7.21 (4.7–10.23)**	**4.78 (2.8–8.75)** ^*∗*^

*Number of antenatal care visits*					
≤4 visits	3433 (72.17%)	103 (2.17%)		1	1
>4 visits	1023 (21.5%)	198 (4.16%)		**6.45 (2.5–9.2)**	**2.85 (1.38–5.73)** ^*∗*^

^*∗*^The variable is significant with a *p* value of less than 0.05.

## Data Availability

All the organized datasets that support the finding of the study are available upon request from the corresponding author.

## Abbreviations

ANC: Antenatal care
CI: Confidence interval
CS: Caesarean section
IBF: Initiate breastfeeding
OR: Odds ratio
UNICEF: United Nations International Children's Emergency Fund
WHO: World Health Organization.
